# Ethyl 6-(4-fluoro­phen­yl)-4-hy­droxy-2-sulfanyl­idene-4-trifluoro­methyl-1,3-diazinane-5-carboxyl­ate

**DOI:** 10.1107/S1600536812007465

**Published:** 2012-02-29

**Authors:** Bao-Jun Huang, Lei Zhu, Qin He

**Affiliations:** a Institute of Surface Micro and Nano Materials, Xuchang University, Xuchang, Henan Province 461000, People’s Republic of China; b College of Chemistry and Chemical Engineering, Xuchang University, Xuchang, Henan Province 461000, People’s Republic of China

## Abstract

In the title compound, C_14_H_14_F_4_N_2_O_3_S, the hexa­hydro­pyrimidine ring adopts a half-chair conformation. The mol­ecular conformation is stabilized by an intra­molecular O—H⋯O hydrogen bond, generating an *S*(6) ring. The crystal structure features O—H⋯S and N—H⋯S hydrogen bonds.

## Related literature

For the bioactivity of dihydro­pyrimidines, see: Atwal *et al.* (1989 ▶); Kappe *et al.* (1997 ▶); Brier *et al.* (2004 ▶); Cochran *et al.* (2005 ▶). For the bioactivity of organofluorine compounds, see: Konz (1997 ▶); Hass (2004 ▶). For a related structure, see: Li *et al.* (2011 ▶).
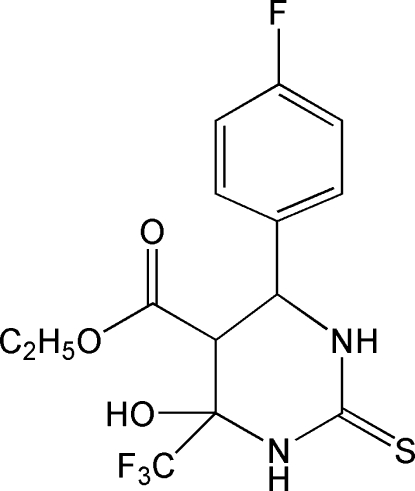


## Experimental

### 

#### Crystal data

C_14_H_14_F_4_N_2_O_3_S
*M* *_r_* = 366.33
Monoclinic, 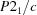

*a* = 11.0091 (12) Å
*b* = 9.9741 (10) Å
*c* = 14.6890 (16) Å
β = 109.269 (12)°
*V* = 1522.6 (3) Å^3^
*Z* = 4
Mo *K*α radiation
μ = 0.27 mm^−1^
*T* = 113 K
0.20 × 0.19 × 0.12 mm

#### Data collection

Rigaku Saturn CCD area-detector diffractometer
Absorption correction: multi-scan (*CrystalClear*; Rigaku, 2009 ▶) *T*_min_ = 0.947, *T*_max_ = 0.968
18960 measured reflections
3627 independent reflections
2979 reflections with *I* > 2σ(*I*)
*R*_int_ = 0.042

#### Refinement

*R*[*F* ^2^ > 2σ(*F* ^2^)] = 0.033
*wR*(*F* ^2^) = 0.084
*S* = 1.01
3627 reflections
227 parameters
H atoms treated by a mixture of independent and constrained refinement
Δρ_max_ = 0.44 e Å^−3^
Δρ_min_ = −0.27 e Å^−3^

### 

Data collection: *CrystalClear* (Rigaku, 2009 ▶); cell refinement: *CrystalClear*; data reduction: *CrystalClear*; program(s) used to solve structure: *SHELXS97* (Sheldrick, 2008 ▶); program(s) used to refine structure: *SHELXL97* (Sheldrick, 2008 ▶); molecular graphics: *SHELXTL* (Sheldrick, 2008 ▶); software used to prepare material for publication: *CrystalStructure* (Rigaku, 2009 ▶).

## Supplementary Material

Crystal structure: contains datablock(s) I, global. DOI: 10.1107/S1600536812007465/fj2501sup1.cif

Structure factors: contains datablock(s) I. DOI: 10.1107/S1600536812007465/fj2501Isup2.hkl

Supplementary material file. DOI: 10.1107/S1600536812007465/fj2501Isup3.cml

Additional supplementary materials:  crystallographic information; 3D view; checkCIF report

## Figures and Tables

**Table 1:** Hydrogen-bond geometry (Å, °)

*D*—H⋯*A*	*D*—H	H⋯*A*	*D*⋯*A*	*D*—H⋯*A*
O1—H1⋯O2	0.84	2.06	2.7767 (13)	144
O1—H1⋯S1^i^	0.84	2.83	3.3796 (10)	124
N1—H1*A*⋯S1^ii^	0.835 (16)	2.635 (17)	3.4566 (12)	168.1 (15)

Symmetry codes: (i) 
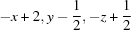
; (ii) 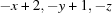.

**Fig. 1.**
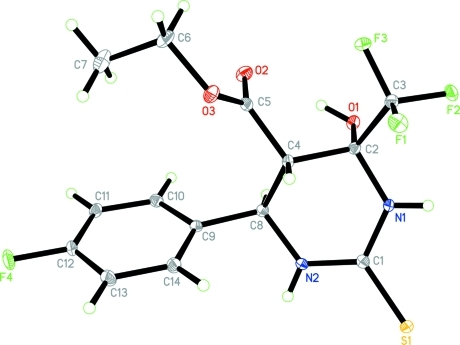
Molecular configuration and atom numbering scheme for (I), with displacement ellipsoids drawn at the 30% probability level.

**Fig. 2.**
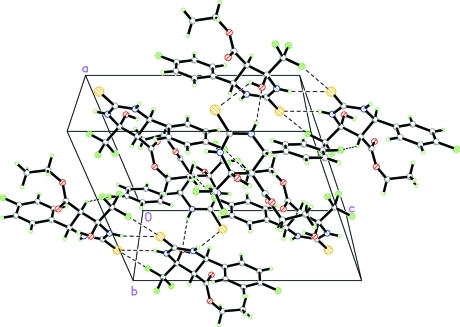
The packing diagram of the title compound. Intermolecular hydrogen bonds are shown as dashed line.

## supplementary crystallographic information

### Comment

Dihydropyrimidine (DHPM) derivatives can be used as potential calcium channel blockers, antihypertensive agents, and α1–1 - a-antagonists (Atwal *et al.*, 1989; Kappe *et al.*, 1997;), inhibitors of mitotic kinesin Eg5 for treating cancer (Cochran *et al.*, 2005; Brier *et al.*, 2004). In addition, compounds that contain fluorine have special bioactivity, *e.g.* flumioxazin is a widely used herbicide (Konz, 1997; Hass, 2004). This led us to focus our attention on the synthesis and bioactivity of these important fused perfluoroalkylated heterocyclic compounds. During the synthesis of DHPM derivatives, the title compound, an intermediate C_14_H_14_F_4_N_2_O_3_S (I) was isolated and the structure confirmed by X-ray diffraction.

In the structure of the title molecule, the hexahydropyrimidine ring adopts a half-chair conformation, the mean planes formed by the ring atoms excluding the C atom bonded to the ethoxy carbonyl group have r.m.s. deviations of 0.0348 Å, the dihedral angle between the mean planes and benzenes ring is 58.18 (5)°. The molecular conformation is stabilized by intramolecular O—H···O hydrogen bond, generating an S(6) ring. The crystal structure is stabilized by intermolecular O—H···S and N—H···S hydrogen bonds. For a crystal structure related to the title compound, see: Li *et al.* (2011).

### Experimental

The title compound was synthesized refluxing for 3 h a stirred solution of 4-fluorobenzaldehyde (2.48 g, 20 mmol), ethyl 4,4,4-trifluoro-3-oxobutanoate(4.42 g, 24 mmol) and thiourea (2.28 g, 30 mmol) in 20 ml of anhydrous ethanol, the reaction catalyzed by sulfamic acid (0.6 g). The solvent was evaporated *in vacuo* and the residue was washed with water. The title compound was recrystallized from 50% aqueous ethanol and single crystals of (I) were obtained by slow evaporation.

### Refinement

Hydrogen atoms involved in hydrogen-bonding inetractions were located by difference methods and their positional and isotropic displacement parameters were refined. Other H atoms were placed in calculated positions, with C—H(aromatic) = 0.95 Å and C—H(aliphatic) = 0.98 Å, 0.99 Å or 1.00 Å, and treated as riding, with *U*_iso_(H) = 1.2*U*eq(C).

### Crystal data

**Table:**

C_14_H_14_F_4_N_2_O_3_S	*F*(000) = 752
*M**_r_* = 366.33	*D*_x_ = 1.598 Mg m^−^^3^
Monoclinic, *P*2_1_/*c*	Mo *K*α radiation, λ = 0.71073 Å
Hall symbol: -P 2ybc	Cell parameters from 5440 reflections
*a* = 11.0091 (12) Å	θ = 2.0–27.9°
*b* = 9.9741 (10) Å	µ = 0.27 mm^−^^1^
*c* = 14.6890 (16) Å	*T* = 113 K
β = 109.269 (12)°	Prism, colorless
*V* = 1522.6 (3) Å^3^	0.20 × 0.19 × 0.12 mm
*Z* = 4	

### Data collection

**Table:**

Rigaku Saturn CCD area-detector diffractometer	3627 independent reflections
Radiation source: rotating anode	2979 reflections with *I* > 2σ(*I*)
Multilayer monochromator	*R*_int_ = 0.042
Detector resolution: 14.63 pixels mm^-1^	θ_max_ = 27.9°, θ_min_ = 2.0°
ω and φ scans	*h* = −14→13
Absorption correction: multi-scan (*CrystalClear*; Rigaku, 2009)	*k* = −13→13
*T*_min_ = 0.947, *T*_max_ = 0.968	*l* = −19→18
18960 measured reflections	

### Refinement

**Table:**

Refinement on *F*^2^	Primary atom site location: structure-invariant direct methods
Least-squares matrix: full	Secondary atom site location: difference Fourier map
*R*[*F*^2^ > 2σ(*F*^2^)] = 0.033	Hydrogen site location: inferred from neighbouring sites
*wR*(*F*^2^) = 0.084	H atoms treated by a mixture of independent and constrained refinement
*S* = 1.01	*w* = 1/[σ^2^(*F*_o_^2^) + (0.050*P*)^2^] where *P* = (*F*_o_^2^ + 2*F*_c_^2^)/3
3627 reflections	(Δ/σ)_max_ = 0.001
227 parameters	Δρ_max_ = 0.44 e Å^−^^3^
0 restraints	Δρ_min_ = −0.27 e Å^−^^3^

### Special details

**Table:**

Geometry. All e.s.d.'s (except the e.s.d. in the dihedral angle between two l.s. planes) are estimated using the full covariance matrix. The cell e.s.d.'s are taken into account individually in the estimation of e.s.d.'s in distances, angles and torsion angles; correlations between e.s.d.'s in cell parameters are only used when they are defined by crystal symmetry. An approximate (isotropic) treatment of cell e.s.d.'s is used for estimating e.s.d.'s involving l.s. planes.
Refinement. Refinement of *F*^2^ against ALL reflections. The weighted *R*-factor *wR* and goodness of fit *S* are based on *F*^2^, conventional *R*-factors *R* are based on *F*, with *F* set to zero for negative *F*^2^. The threshold expression of *F*^2^ > σ(*F*^2^) is used only for calculating *R*-factors(gt) *etc*. and is not relevant to the choice of reflections for refinement. *R*-factors based on *F*^2^ are statistically about twice as large as those based on *F*, and *R*- factors based on ALL data will be even larger.

### Fractional atomic coordinates and isotropic or equivalent isotropic displacement parameters (Å^2^)

**Table:**

	*x*	*y*	*z*	*U*_iso_*/*U*_eq_	
S1	1.09867 (3)	0.59272 (4)	0.13111 (2)	0.01692 (10)	
F1	0.63627 (8)	0.49595 (8)	−0.01023 (6)	0.0223 (2)	
F2	0.70354 (8)	0.30439 (8)	−0.04289 (6)	0.0208 (2)	
F3	0.56928 (7)	0.31300 (9)	0.03576 (6)	0.0211 (2)	
F4	0.77930 (9)	0.74276 (9)	0.59153 (6)	0.0287 (2)	
O1	0.81420 (9)	0.24372 (9)	0.15081 (7)	0.0161 (2)	
H1	0.7778	0.2236	0.1910	0.024*	
O2	0.64286 (9)	0.28689 (10)	0.25049 (7)	0.0214 (2)	
O3	0.55299 (9)	0.49304 (10)	0.22282 (7)	0.0200 (2)	
N1	0.89236 (10)	0.43819 (12)	0.10096 (8)	0.0139 (2)	
N2	0.96704 (11)	0.54349 (13)	0.24822 (8)	0.0156 (2)	
C1	0.97865 (12)	0.52093 (13)	0.16191 (9)	0.0138 (3)	
C2	0.78397 (12)	0.37743 (13)	0.12011 (9)	0.0131 (3)	
C3	0.67215 (13)	0.37300 (14)	0.02481 (10)	0.0159 (3)	
C4	0.74924 (12)	0.46406 (13)	0.19506 (9)	0.0132 (3)	
H4	0.7220	0.5551	0.1673	0.016*	
C5	0.64259 (13)	0.40290 (14)	0.22563 (9)	0.0154 (3)	
C6	0.44550 (14)	0.44909 (18)	0.25374 (11)	0.0270 (4)	
H6A	0.4297	0.3523	0.2400	0.032*	
H6B	0.3666	0.4985	0.2169	0.032*	
C7	0.47442 (16)	0.47361 (19)	0.35960 (12)	0.0333 (4)	
H7A	0.5492	0.4200	0.3963	0.050*	
H7B	0.3998	0.4479	0.3781	0.050*	
H7C	0.4931	0.5689	0.3736	0.050*	
C8	0.87196 (12)	0.47696 (14)	0.28305 (9)	0.0138 (3)	
H8	0.9039	0.3849	0.3058	0.017*	
C9	0.85100 (12)	0.55238 (14)	0.36593 (9)	0.0136 (3)	
C10	0.84025 (13)	0.47892 (14)	0.44373 (10)	0.0160 (3)	
H10	0.8506	0.3843	0.4449	0.019*	
C11	0.81454 (13)	0.54231 (15)	0.51951 (10)	0.0174 (3)	
H11	0.8055	0.4924	0.5720	0.021*	
C12	0.80260 (13)	0.67954 (15)	0.51632 (10)	0.0183 (3)	
C13	0.81342 (14)	0.75668 (15)	0.44131 (10)	0.0216 (3)	
H13	0.8051	0.8515	0.4417	0.026*	
C14	0.83700 (14)	0.69099 (14)	0.36509 (10)	0.0184 (3)	
H14	0.8436	0.7414	0.3120	0.022*	
H2A	1.0172 (14)	0.5927 (16)	0.2825 (11)	0.015 (4)*	
H1A	0.9054 (15)	0.4242 (17)	0.0489 (12)	0.026 (5)*	

### Atomic displacement parameters (Å^2^)

**Table:**

	*U*^11^	*U*^22^	*U*^33^	*U*^12^	*U*^13^	*U*^23^
S1	0.01646 (18)	0.01854 (18)	0.01832 (18)	−0.00502 (13)	0.00919 (14)	−0.00420 (14)
F1	0.0232 (4)	0.0197 (5)	0.0198 (4)	0.0034 (3)	0.0016 (3)	0.0036 (3)
F2	0.0202 (4)	0.0267 (5)	0.0166 (4)	−0.0024 (3)	0.0075 (3)	−0.0088 (3)
F3	0.0137 (4)	0.0288 (5)	0.0210 (4)	−0.0066 (3)	0.0061 (3)	−0.0033 (4)
F4	0.0441 (6)	0.0261 (5)	0.0230 (5)	−0.0029 (4)	0.0206 (4)	−0.0098 (4)
O1	0.0198 (5)	0.0124 (5)	0.0183 (5)	0.0014 (4)	0.0093 (4)	0.0014 (4)
O2	0.0211 (5)	0.0190 (5)	0.0266 (6)	−0.0008 (4)	0.0114 (4)	0.0025 (4)
O3	0.0160 (5)	0.0229 (6)	0.0235 (5)	0.0035 (4)	0.0097 (4)	0.0004 (4)
N1	0.0138 (6)	0.0172 (6)	0.0128 (6)	−0.0028 (4)	0.0071 (5)	−0.0034 (5)
N2	0.0146 (6)	0.0196 (6)	0.0125 (6)	−0.0063 (5)	0.0044 (5)	−0.0045 (5)
C1	0.0134 (7)	0.0128 (6)	0.0149 (7)	0.0019 (5)	0.0044 (5)	−0.0002 (5)
C2	0.0131 (6)	0.0126 (7)	0.0148 (6)	−0.0001 (5)	0.0061 (5)	−0.0005 (5)
C3	0.0157 (7)	0.0160 (7)	0.0178 (7)	−0.0005 (5)	0.0078 (6)	−0.0022 (5)
C4	0.0141 (7)	0.0137 (6)	0.0124 (6)	0.0007 (5)	0.0054 (5)	−0.0006 (5)
C5	0.0143 (7)	0.0185 (7)	0.0127 (6)	−0.0006 (5)	0.0034 (5)	−0.0025 (5)
C6	0.0152 (7)	0.0367 (9)	0.0330 (9)	0.0005 (6)	0.0133 (7)	−0.0020 (7)
C7	0.0292 (9)	0.0453 (11)	0.0317 (9)	0.0066 (8)	0.0185 (8)	0.0055 (8)
C8	0.0134 (6)	0.0149 (7)	0.0134 (6)	−0.0005 (5)	0.0049 (5)	0.0000 (5)
C9	0.0109 (6)	0.0158 (7)	0.0133 (6)	−0.0016 (5)	0.0028 (5)	−0.0013 (5)
C10	0.0170 (7)	0.0141 (7)	0.0166 (7)	−0.0004 (5)	0.0051 (5)	0.0002 (5)
C11	0.0181 (7)	0.0222 (7)	0.0129 (6)	−0.0017 (6)	0.0064 (6)	0.0023 (6)
C12	0.0202 (7)	0.0217 (8)	0.0152 (7)	−0.0024 (6)	0.0092 (6)	−0.0064 (6)
C13	0.0294 (8)	0.0133 (7)	0.0243 (8)	−0.0019 (6)	0.0119 (6)	−0.0028 (6)
C14	0.0242 (8)	0.0161 (7)	0.0169 (7)	−0.0020 (6)	0.0097 (6)	0.0009 (6)

### Geometric parameters (Å, º)

**Table:**

S1—C1	1.6905 (13)	C4—H4	1.0000
F1—C3	1.3381 (16)	C6—C7	1.501 (2)
F2—C3	1.3428 (15)	C6—H6A	0.9900
F3—C3	1.3367 (15)	C6—H6B	0.9900
F4—C12	1.3678 (15)	C7—H7A	0.9800
O1—C2	1.4120 (15)	C7—H7B	0.9800
O1—H1	0.8400	C7—H7C	0.9800
O2—C5	1.2130 (16)	C8—C9	1.5122 (18)
O3—C5	1.3252 (16)	C8—H8	1.0000
O3—C6	1.4675 (16)	C9—C14	1.3906 (19)
N1—C1	1.3504 (17)	C9—C10	1.3950 (18)
N1—C2	1.4462 (16)	C10—C11	1.3881 (19)
N1—H1A	0.835 (16)	C10—H10	0.9500
N2—C1	1.3345 (17)	C11—C12	1.374 (2)
N2—C8	1.4668 (16)	C11—H11	0.9500
N2—H2A	0.785 (16)	C12—C13	1.381 (2)
C2—C3	1.5296 (18)	C13—C14	1.3936 (19)
C2—C4	1.5436 (17)	C13—H13	0.9500
C4—C5	1.5167 (18)	C14—H14	0.9500
C4—C8	1.5363 (18)		
			
C2—O1—H1	109.5	C7—C6—H6A	109.5
C5—O3—C6	117.11 (11)	O3—C6—H6B	109.5
C1—N1—C2	124.90 (11)	C7—C6—H6B	109.5
C1—N1—H1A	114.4 (11)	H6A—C6—H6B	108.1
C2—N1—H1A	120.7 (11)	C6—C7—H7A	109.5
C1—N2—C8	123.93 (12)	C6—C7—H7B	109.5
C1—N2—H2A	116.6 (10)	H7A—C7—H7B	109.5
C8—N2—H2A	119.4 (10)	C6—C7—H7C	109.5
N2—C1—N1	117.77 (12)	H7A—C7—H7C	109.5
N2—C1—S1	120.74 (10)	H7B—C7—H7C	109.5
N1—C1—S1	121.49 (10)	N2—C8—C9	111.83 (11)
O1—C2—N1	109.59 (10)	N2—C8—C4	105.97 (10)
O1—C2—C3	107.38 (11)	C9—C8—C4	113.17 (10)
N1—C2—C3	107.50 (10)	N2—C8—H8	108.6
O1—C2—C4	112.87 (10)	C9—C8—H8	108.6
N1—C2—C4	108.58 (11)	C4—C8—H8	108.6
C3—C2—C4	110.79 (10)	C14—C9—C10	119.37 (13)
F3—C3—F1	107.59 (10)	C14—C9—C8	122.23 (12)
F3—C3—F2	107.24 (11)	C10—C9—C8	118.35 (12)
F1—C3—F2	107.32 (11)	C11—C10—C9	120.83 (13)
F3—C3—C2	111.15 (10)	C11—C10—H10	119.6
F1—C3—C2	111.82 (11)	C9—C10—H10	119.6
F2—C3—C2	111.49 (10)	C12—C11—C10	117.96 (13)
C5—C4—C8	109.66 (10)	C12—C11—H11	121.0
C5—C4—C2	112.60 (11)	C10—C11—H11	121.0
C8—C4—C2	106.91 (10)	F4—C12—C11	118.22 (12)
C5—C4—H4	109.2	F4—C12—C13	118.43 (13)
C8—C4—H4	109.2	C11—C12—C13	123.34 (13)
C2—C4—H4	109.2	C12—C13—C14	117.84 (14)
O2—C5—O3	125.76 (12)	C12—C13—H13	121.1
O2—C5—C4	123.20 (12)	C14—C13—H13	121.1
O3—C5—C4	111.03 (11)	C9—C14—C13	120.64 (13)
O3—C6—C7	110.79 (13)	C9—C14—H14	119.7
O3—C6—H6A	109.5	C13—C14—H14	119.7
			
C8—N2—C1—N1	4.3 (2)	C2—C4—C5—O2	49.06 (18)
C8—N2—C1—S1	−175.06 (10)	C8—C4—C5—O3	109.10 (13)
C2—N1—C1—N2	3.4 (2)	C2—C4—C5—O3	−132.01 (12)
C2—N1—C1—S1	−177.24 (10)	C5—O3—C6—C7	91.06 (16)
C1—N1—C2—O1	−99.95 (14)	C1—N2—C8—C9	−161.16 (12)
C1—N1—C2—C3	143.65 (13)	C1—N2—C8—C4	−37.41 (17)
C1—N1—C2—C4	23.75 (17)	C5—C4—C8—N2	−176.87 (11)
O1—C2—C3—F3	59.68 (13)	C2—C4—C8—N2	60.79 (13)
N1—C2—C3—F3	177.53 (10)	C5—C4—C8—C9	−53.96 (15)
C4—C2—C3—F3	−63.98 (14)	C2—C4—C8—C9	−176.31 (10)
O1—C2—C3—F1	179.95 (10)	N2—C8—C9—C14	44.36 (17)
N1—C2—C3—F1	−62.21 (13)	C4—C8—C9—C14	−75.23 (16)
C4—C2—C3—F1	56.28 (14)	N2—C8—C9—C10	−138.31 (12)
O1—C2—C3—F2	−59.90 (13)	C4—C8—C9—C10	102.10 (14)
N1—C2—C3—F2	57.94 (14)	C14—C9—C10—C11	0.66 (19)
C4—C2—C3—F2	176.43 (10)	C8—C9—C10—C11	−176.75 (12)
O1—C2—C4—C5	−54.01 (14)	C9—C10—C11—C12	−1.3 (2)
N1—C2—C4—C5	−175.72 (10)	C10—C11—C12—F4	−178.98 (12)
C3—C2—C4—C5	66.45 (14)	C10—C11—C12—C13	0.8 (2)
O1—C2—C4—C8	66.48 (13)	F4—C12—C13—C14	−179.92 (13)
N1—C2—C4—C8	−55.23 (13)	C11—C12—C13—C14	0.3 (2)
C3—C2—C4—C8	−173.07 (10)	C10—C9—C14—C13	0.5 (2)
C6—O3—C5—O2	0.9 (2)	C8—C9—C14—C13	177.79 (13)
C6—O3—C5—C4	−178.03 (11)	C12—C13—C14—C9	−1.0 (2)
C8—C4—C5—O2	−69.83 (16)		

### Hydrogen-bond geometry (Å, º)

**Table:**

*D*—H···*A*	*D*—H	H···*A*	*D*···*A*	*D*—H···*A*
O1—H1···O2	0.84	2.06	2.7767 (13)	144
O1—H1···S1^i^	0.84	2.83	3.3796 (10)	124
N1—H1*A*···S1^ii^	0.835 (16)	2.635 (17)	3.4566 (12)	168.1 (15)

Symmetry codes: (i) −*x*+2, *y*−1/2, −*z*+1/2; (ii) −*x*+2, −*y*+1, −*z*.
